# Minimally invasive mitral valve surgery in a middle-income country: feasibility and learning curve in a low-volume center

**DOI:** 10.1186/s13019-026-03879-3

**Published:** 2026-02-28

**Authors:** Simón Muñoz González, Susana Cardona Bernal, Maria Eugenia Echavarría, Sofía Sierra Hernández, Jonathan Alexis Sánchez Zapata, Alejandro Escobar Uribe, Rafael José Meza Jiménez

**Affiliations:** 1 Universidad de Antioquia, Cra. 51D #62 − 29, La Candelaria, Medellín, Colombia; 2 Universidad CES, Medellín, Colombia; 3 Somer Incare, Medellín, Colombia

**Keywords:** Mitral valve, Mitral valve surgery, Minimally invasive cardiac surgery, Learning curve

## Abstract

**Introduction:**

Minimally invasive mitral valve surgery (MIM-VS) has emerged as a safe alternative to conventional median sternotomy (MS), offering advantages such as faster recovery and improved cosmetic results. However, evidence regarding its implementation and learning curve in low-volume centers within middle-income countries remains limited. This study aimed to compare the clinical outcomes of MIM-VS and MS in a low-volume center in Colombia and to assess the institutional learning curve.

**Methodology:**

A retrospective study was conducted on 112 patients who underwent mitral valve surgery via MIM-VS (*n* = 63) or MS (*n* = 49) between 2015 and mid-2024. A 1:1 nearest-neighbor propensity score matching algorithm without replacement was used to minimize confounding variables. Early postoperative outcomes were compared, and the learning curve for MIM-VS was assessed using cumulative sum (CUSUM) analysis.

**Results:**

After matching, outcomes were comparable between groups. MIM-VS was associated with longer cardiopulmonary bypass (125.0 vs. 77.0 min; *p* < 0.001) and aortic cross-clamp times (93.0 vs. 59.0 min; *p* < 0.001). However, patients in the MIM-VS group exhibited shorter ICU stays (1.0–3.0 vs. 2.0–3.0 days; *p* = 0.045). Complication rates, including mortality, paravalvular leak, and femoral vessel injury, were similar between groups. CUSUM analysis showed an initial learning phase characterized by variable operative times, which stabilized after 40 cases. Proficiency was reached after 50 cases, correlating with reduced operative times and better outcomes. Success and failure rates improved, stabilizing after case 23, reinforcing the need for structured training and experience.

**Conclusion:**

The implementation of MIM-VS in a low-volume center in a middle-income country is feasible, safe and non-inferior to MS. Although initially associated with prolonged operative times, procedural efficiency improves with experience without compromising patient safety. These findings support the structured adoption of minimally invasive techniques in similar resource-constrained settings committed to training and quality monitoring.

**Supplementary Information:**

The online version contains supplementary material available at 10.1186/s13019-026-03879-3.

## Introduction

Since the mid-1990s, minimally invasive mitral valve surgery (MIM-VS) has progressively gained acceptance, demonstrating clear advantages compared with conventional surgery via median sternotomy (MS) [1–3]. Documented benefits include reduced postoperative morbidity, less pain, decreased need for blood transfusions, shorter hospital stays, faster recovery, and improved aesthetic outcomes [2–7]. Currently, MIM-VS is predominantly performed via a right mini-thoracotomy approach utilizing peripheral cannulation for cardiopulmonary bypass [7, 8].

Notwithstanding these documented benefits, widespread adoption presents challenges, particularly during the early phase of the learning curve. These challenges arise from the need for specific training in these techniques, the use of specialized instrumentation, and the increased cardiopulmonary bypass (CPB) and aortic cross-clamping times [7, 9]. Furthermore, potential complications, such as cerebrovascular events, may increase procedural complexity [10, 11]. Consequently, successful implementation has been largely restricted to high-volume centers where surgeons can overcome the learning curve through frequent practice [2, 4, 7, 9]. This volume-dependent limitation has created a gap in the literature, as research supporting the establishment of MIM-VS in low-volume centers remains limited [12, 13]. Moreover, data regarding the clinical performance of advanced cardiac techniques in middle-income countries are scarce, where unique hurdles such as resource disparities and structural deficiencies persist.

To ensure a rigorous evaluation of clinical outcomes despite these challenges, comparative studies increasingly use propensity score analyses to account for selection bias and validate safety [2, 7, 9, 14]. Similarly, analyzing the learning curve using the cumulative sum (CUSUM) method has allowed for evaluating surgical performance trends and determining the number of procedures required to optimize clinical outcomes [15, 16]. Various studies have demonstrated that as surgical teams gain experience, there is a progressive reduction in operative times and a lower incidence of complications, highlighting the importance of structured training strategies and continuous performance monitoring [15–17].

Expanding the body of available data in these settings is essential to promote the feasibility, reproducibility, and safe adoption of minimally invasive approaches. Therefore, the objective of this study was to evaluate the clinical outcomes of patients treated at a low-volume center in Colombia who underwent mitral valve surgery via both conventional and minimally invasive techniques. Additionally, a CUSUM analysis was conducted to determine the institutional learning curve.

## Materials and methods

### Study design

This retrospective study analyzes the clinical outcomes of a patient cohort treated at a low-volume center within a specialized cardiovascular clinic in Colombia. Data were collected from electronic medical records spanning 2015 to mid-2024.

### Study population and decision-making process

The study included adult patients aged 18 years or older who underwent mitral valve surgery (replacement or valvuloplasty) via a minimally invasive approach or MS at a low-volume center. Patients with complete medical records were included. Exclusion criteria comprised prior valve surgery, a history of a mitral device, concomitant valve procedures or coronary artery bypass graft (CABG), urgent surgery, or orotracheal intubation prior to entering the operating room.

The selection of candidates for MIM-VS was based on a multidimensional evaluation that considered anatomy, comorbidities, and technical feasibility, within the context of a low-volume center in a consolidation phase. Priority was given to patients with isolated mitral pathology, primarily degenerative mitral regurgitation, who presented with favorable anatomy for peripheral cannulation and had no contraindications to right lateral access. In contrast, patients requiring concomitant procedures (such as coronary artery bypass or multivalvular surgery), or those with limited femoral access, severe chest wall deformities, or extensive aortic calcification, were allocated to conventional MS. With respect to arrhythmia management, due to the unavailability of minimally invasive ablation devices at our institution, patients with atrial fibrillation (AF) or severe left atrial dilation requiring surgical ablation were also assigned to the sternotomy group to ensure comprehensive treatment.

### Minimally invasive intervention and conventional technique

The minimally invasive approach was performed in all patients via a right anterolateral minithoracotomy, with cardiopulmonary bypass using peripheral cannulation (femoral venous and arterial). Patients with a history of AF were excluded from MIM-VS due to the requirement for a concomitant Maze procedure, which necessitates a traditional approach. Aortic cross-clamping was performed transtoracically through a small additional incision in the right axillary area. No patients underwent video-assisted techniques. Conventional mitral valve surgery was performed via median sternotomy with central cannulation for cardiopulmonary bypass.

### Outcome description

This study assessed early outcomes, defined as those occurring during the postoperative hospital stay. The outcomes were intraoperative conversion to median sternotomy for any reason, aortic cross-clamp time, cardiopulmonary bypass time, intraoperative blood product utilization, intraoperative mortality, in-hospital mortality, reoperation for any cause, sustained arrhythmia during postoperative hospitalization, femoral vessel injury, intensive care unit (ICU) length of stay, hospital length of stay, orotracheal intubation time, surgical site infection during hospitalization, moderate or severe paravalvular leak (identified via intraoperative transesophageal echocardiography [TEE] after valve replacement), and moderate or severe mitral regurgitation (identified via intraoperative TEE after valve repair).

### Data collection

A protected database was created using Microsoft Excel^®^ (version 16.95.1) based on patient medical records. This database included general patient information such as age, sex, body mass index (BMI), preoperative left ventricular ejection fraction, and medical history of hypertension, chronic obstructive pulmonary disease (COPD), AF, diabetes mellitus, and smoking status. Data collection was performed by the main investigators following approval from the institutional ethics committee.

### Statistical analysis

Continuous variables were reported as mean ± standard deviation or median (interquartile range [IQR]), while categorical variables were expressed as frequencies and percentages. The Mann-Whitney U test was used to compare continuous variables. Associations between categorical variables were assessed using the chi-square test, and Fisher’s exact test was applied when cell counts were below five. All comparisons were two-tailed, and a *p*-value < 0.05 was considered statistically significant.

To minimize selection bias and potential confounding effects, a propensity score matching model was implemented. Individual propensity scores were estimated using a logistic regression model incorporating four baseline covariates: sex, age, BMI, and primary diagnosis. These variables were selected to account for demographic and clinical differences between the surgical groups. Matching was performed using 1:1 nearest-neighbor matching without replacement. The quality of matching was assessed by evaluating the balance in the distribution of patient characteristics using standardized mean differences (SMDs). Baseline surgical characteristics and outcomes were analyzed both before and after matching. Additionally, a Pearson chi-square test was conducted to assess the association between AF history and study outcomes. All statistical analyses were performed using IBM SPSS Statistics for Windows, version 25.0 (IBM Corp., Armonk, NY, USA).

### Learning curve analysis using CUSUM

A CUSUM analysis was performed to assess the learning curve for MIM-VS. The statistical principles outlined in Novick’s methodology [18], were applied, defining CUSUM as Sn = ∑(Xi − p0), where Xi = 0 denotes a successful case and Xi = 1 denotes a failed case. In this study, p0​ was set at 0.1, indicating an acceptable composite failure rate of 10%. For the failure analysis, boundary lines were established following Novick’s methodology, setting an 80% alert threshold and a 95% alarm threshold [18].

Based on previous studies on minimally invasive mitral valve surgery, a “failed case” was defined by the occurrence of any of the following outcomes: intraoperative mortality, in-hospital mortality, reoperation for any cause, femoral vessel injury, moderate or severe paravalvular leak, moderate or severe mitral regurgitation, conversion to median sternotomy, and surgical site infection. Additionally, a CUSUM analysis was used to evaluate variations in CPB time, aortic cross-clamp time, and hospital length of stay by calculating the difference between each chronologically ordered case’s procedural time and the overall mean procedural time. In the CUSUM method, an upward trend in the curve indicates accumulated performance that is worse than the expected reference value, whereas a downward slope reflects performance that is better than expected. Stabilization or flattening of the curve denotes consistency and reduced variability in operative metrics. All statistical computations and visualizations of the learning curves were conducted using R software, version 4.4.3 (R Foundation for Statistical Computing, Vienna, Austria).

### Ethical considerations

Data collection was conducted retrospectively, adhering to the guidelines of the Declaration of Helsinki from the 75th General Assembly in 2024 [19]. According to current national regulations, this study is classified as risk-free since it is based solely on retrospective medical record review. The study protocol was approved by the institutional ethics committee.

## Results

### Baseline characteristics of the study population

A total of 227 medical records of patients who underwent MS and MIM-VS were reviewed. Of these, 115 patients were excluded based on the exclusion criteria. According to the inclusion criteria, 112 patients were evaluated, 63 of whom (56.3%) underwent mitral valve surgery via MIM-VS, while 49 (43.7%) underwent surgery through the conventional MS approach. The baseline characteristics of the overall study population and the propensity score matching are summarized in Table 1.

In the unmatched cohort, significant differences were observed in sex (*p* = 0.004), weight (*p* = 0.008), diagnosis distribution (*p* = 0.007), and the prevalence of AF (*p* < 0.001). Following propensity score matching, 45 patients were included in each group. The matched cohort showed excellent balance across demographics and comorbidities, with no statistically significant differences observed between the surgical approaches. The improvement in covariate balance is substantiated by the Love plot (Supplementary file 1) and the propensity score distribution (Supplementary file 2), which confirm that SMDs were reduced to negligible levels and that adequate overlap between groups was achieved.

The only exception was the history of AF, which remained significantly more prevalent in the MS group (*p* < 0.001). This imbalance reflects the institutional protocol described in the Methods, whereby patients requiring surgical ablation were allocated to MS.

**Table 1:** Baseline characteristics of the population (unmatched and matched cohorts)

Variable	Unmatched cohort				Matched cohort			
	Sugical approach				Sugical approach			
	Total	MS *N* = 49	MIM-VS *N* = 63	*p*-value	Total	MS *N* = 45	MIM-VS *N* = 45	*p*-value
**Demographics & Anthropometrics**								
Female, n (%)	58 (51.8)	33 (67.3)	25 (39.7)	**0.004**	53 (58.9)	29 (64.4)	24 (53.3)	0.284
Male, n (%)	54 (48.2)	16 (32.7)	38 (60.3)		37 (41.1)	16 (35.6)	21 (46.7)	
Age, median [IQR]	57 [46–66]	56 [46–65]	57 [47–66]	0.995	58 [46–67]	56 [39–66]	60 [50–67]	0.583
Weight (kg), median [IQR]	65 [56–73]	61.5 [55–67]	69 [58–79]	**0.008**	65 [56–71]	62 [56–67]	65 [55–73]	0.273
Height (m), median [IQR]	1.7 [1.6–1.7]	1.6 [1.6–1.7]	1.7 [1.6–1.8]	0.709	1.6 [1.6–1.7]	1.6 [1.6–1.7]	1.6 [1.6–1.7]	0.510
BMI (kg/m^2^), median [IQR]	24.7 [21.4–27.5]	23.0 [20.7–25.7]	25.3 [21.8–27.7]	0.315	24.4 [21.5–27.2]	23.8 [20.9–26.3]	25.0 [21.6–27.5]	0.503
**Medical history**,** n (%)**								
Hypertension	47 (42.0)	17 (34.7)	30 (47.6)	0.183	38 (42.2)	15 (33.3)	23 (51.1)	0.088
AF	27 (24.1)	26 (53.1)	1 (1.6)	**0.000**	22 (24.4)	22 (48.9)	0 (0.0)	**0.000**
COPD	3 (2.7)	2 (4.1)	1 (1.6)	0.580	3 (3.3)	2 (4.4)	1 (2.2)	0.557
Diabetes Mellitus	4 (3.6)	1 (2.0)	3 (4.8)	0.630	4 (4.4)	1 (2.2)	3 (6.7)	0.306
Smoking	29 (25.9)	15 (30.6)	14 (22.2)	0.386	24 (26.7)	15 (3.3)	9 (20.0)	0.153
Valve surgery	1 (0.9)	1 (2.0)	0 (0.0)	0.438	1 (1.1)	1 (2.2)	0 (0.0)	0.315
Mitral device	1 (0.9)	1 (2.0)	0 (0.0)	0.438	1 (1.1)	1 (2.2)	0 (0.0)	0.315
**Diagnosis**								
Double lesion	10 (8.9)	6 (12.2)	4 (6.3)	**0.007**	10 (11.1)	6 (13.3)	4 (8.9)	0.282
Stenosis	9 (8.0)	8 (16.3)	1 (1.6)		5 (5.6)	4 (8.9)	1 (2.2)	
Regurgitation	93 (83.0)	35 (71.4)	58 (92.1)		75 (83.3)	35 (77.8)	40 (88.9)	
**Echocardiographic data**								
Preoperative LVEF, median [IQR]	58 [53.5–65.0]	58.0 [50.0–65.0]	58.0 [54.0–65.0]	0.216	58.0 [53.0–65.0]	58.0 [49.0–65.0]	58.0 [55.0–65.0]	0.399

MS: median sternotomy; MIM-VS: minimally invasive mitral valve surgery; BMI: body mass index; AF: atrial fibrillation; COPD: chronic obstructive pulmonary disease; LVEF: left ventricle ejection fraction; IQR: Interquartile Range

### Surgical characteristics

Operative data and valve specifications are detailed in Table 2. In the unmatched cohort, valve replacement was the predominant procedure overall. However, the MIM-VS group demonstrated a significantly higher rate of mitral valve repair compared to the MS group (*p* = 0.024). Regarding prosthesis selection, mechanical valves were more frequently implanted in the MS group, while biological valves were similarly distributed between approaches (*p* = 0.021). Notably, these significant differences, specifically the higher frequency of repair in the MIM-VS group and the greater use of mechanical valves in the MS group, persisted after propensity score matching (*p* = 0.048 and *p* = 0.037, respectively). No significant differences were observed in the type of cardioplegia used.

**Table 2:** Surgical characteristics

**Unmatched cohort**									**Matched cohort**						
**Variable**		**Surgical approach**							**Surgical approach**						
		**Total**		**MS ** **N = 49**		**MIM-VS** **N = 63**			**Total**		**MS** **N = 45**		**MIM-VS** **N = 45**		
		n	%	n	%	n	%	*p* value	n	%	n	%	n	%	*p* value
Procedure type	Valve replacement	92	82.0%	45	91.8%	47	74.6%	**0.024**	75	83.3%	41	91.1%	34	75.6%	**0.048**
	Valve repair (valvuloplasty)	20	17.9%	4	8.2%	16	25.4%		15	16.7%	4	8.9%	11	24.4%	
Cardioplegia type	Custodiol	16	14.3%	5	10.2%	11	17.5%	0.415	13	14.4%	4	8.9%	9	20.0%	0.134
	Del Nido	96	85.7%	44	89.8%	52	82.5%		77	85.6%	41	91.1%	36	80.0%	
Valve type	NA	20	17.9%	4	8.2%	16	25.4%	**0.021**	15	16.7%	4	8.9%	11	24.4%	**0.037**
	Biological	60	53.6%	26	53.1%	34	54.0%		50	55.6%	24	53.3%	26	57.8%	
	Mechanical	32	28.6%	19	38.8%	13	20.6%		25	27.8%	17	37.8%	8	17.8%	

MS: median sternotomy; MIM-VS: minimally invasive mitral valve surgery; NA: not applicable

### Outcomes

Surgical outcomes for both cohorts are presented in Table 3. As expected, operative times—specifically cardiopulmonary bypass and aortic cross-clamp duration—were significantly prolonged in the MIM-VS group compared to the MS (*p* < 0.001). Regarding safety endpoints, intraoperative conversion to sternotomy in the matched MIM-VS group was low (*n* = 1), with no significant difference compared to the MS group (*p* = 0.315). Similarly, no statistically significant differences were observed in intraoperative mortality, reoperation for any cause, femoral vessel injury, surgical site infection, or in-hospital mortality.

With respect to postoperative recovery, the MIM-VS group demonstrated a statistically significant reduction in ICU length of stay (*p* = 0.045). Although overall hospital length of stay did not reach statistical significance (*p* = 0.057), a trend toward shorter hospitalization was observed in the minimally invasive cohort.

**Table 3:** Surgical outcomes: unmatched and matched cohorts

Variable	Unmatched cohort				Matched cohort			
	Sugical approach				Sugical approach			
	Total	MS *N* = 49	MIM-VS *N* = 63	*p*-value	Total	MS *N* = 45	MIM-VS *N* = 45	*p*-value
**Intraperative data**								
CBP time (min), median [IQR]	104.0 [80.0–134.5.0.5]	80.0 [70–93]	13.0 [105–149]	**< 0.001**	97.0 [77–126]	77.0 [69–93]	125.0 [100–147]	**< 0.001**
Cross-clamp time (min), median [IQR]	72.5 [58–100]	60.0 [47–67]	95.0 [72–106]	**< 0.001**	69.0 [55–98]	59.0 [47–67]	93.0 [71–106]	**< 0.001**
Conversion to MS, n (%)	3 (2.7)	0 (0.0)	3 (4.8)	**< 0.001**	1 (1.1)	0 (0.0)	1 (2.2)	0.315
Blood transfusion, n (%)	56 (50.0)	27 (55.1)	29 (46.0)	0.446	47 (52.2)	23 (51.1)	24 (53.3)	0.833
Intraoperative death, n (%)	1 (0.9)	1 (2.0)	0 (0.0)	0.438	1 (1.1)	1 (2.2)	0 (0.0)	0.315
**Postoperative data**								
ICU stay (days), median [IQR]	2.0 [1–3]	3.0 [2–4]	2.0 [1–3]	0.056	2.0 [1–3]	2.0 [2–3]	2.0 [1–3]	**0.045**
Hospital stay (days from surgery), median [IQR]	11.0 [7.0–14.5.0.5]	12.0 [9–17]	9.0 [6–14]	0.411	11.0 [8–15]	12.0 [9–16]	9.0 [7–14]	0.057
Orotracheal intubation time (days), median [IQR]	0.0 [0–1]	1.0 [0–1]	0.0 [0–1]	0.295	0.0 [0–1]	1.0 [0–1]	0.0 [0–1]	0.311
Paravalvular leak (moderate or severe)	0 (0.0)	0 (0.0)	0 (0.0)	0.063	0 (0.0)	0 (0.0)	0 (0.0)	0.114
Mitral valve regurgitation (moderate or severe)	3 (2.7)	0 (0.0)	3 (4.8)	**0.028**	2 (2.2)	0 (0.0)	2 (4.4)	0.077
Reoperation for any cause	13 (11.6)	6 (12.2)	7 (11.1)	1.000	9 (10.0)	5 (11.1)	4 (8.9)	0.725
Postoperative arrythmia	25 (22.3)	6 (12.2)	19 (30.2)	**0.038**	19 (21.1)	6 (13.3)	13 (28.9)	0.071
Femoral vessel injury	2 (1.8)	0 (0.0)	2 (3.2)	0.208	2 (2.2)	0 (0.0)	2 (4.4)	0.153
Surgical site infection	2 (1.8)	0 (0.0)	2 (3.2)	0.503	2 (2.2)	0 (0.0)	2 (4.4)	0.153
In-hospital mortality	8 (7.1)	4 (8.2)	4 (6.3)	0.728	8 (8.9)	4 (8.9)	4 (8.9)	1.000

MS: median sternotomy; MIM-VS: minimally invasive mitral valve surgery; ICU: intensive care unit; IQR: Interquartile Range

### CUSUM analysis of learning curve

**Fig. 1:**
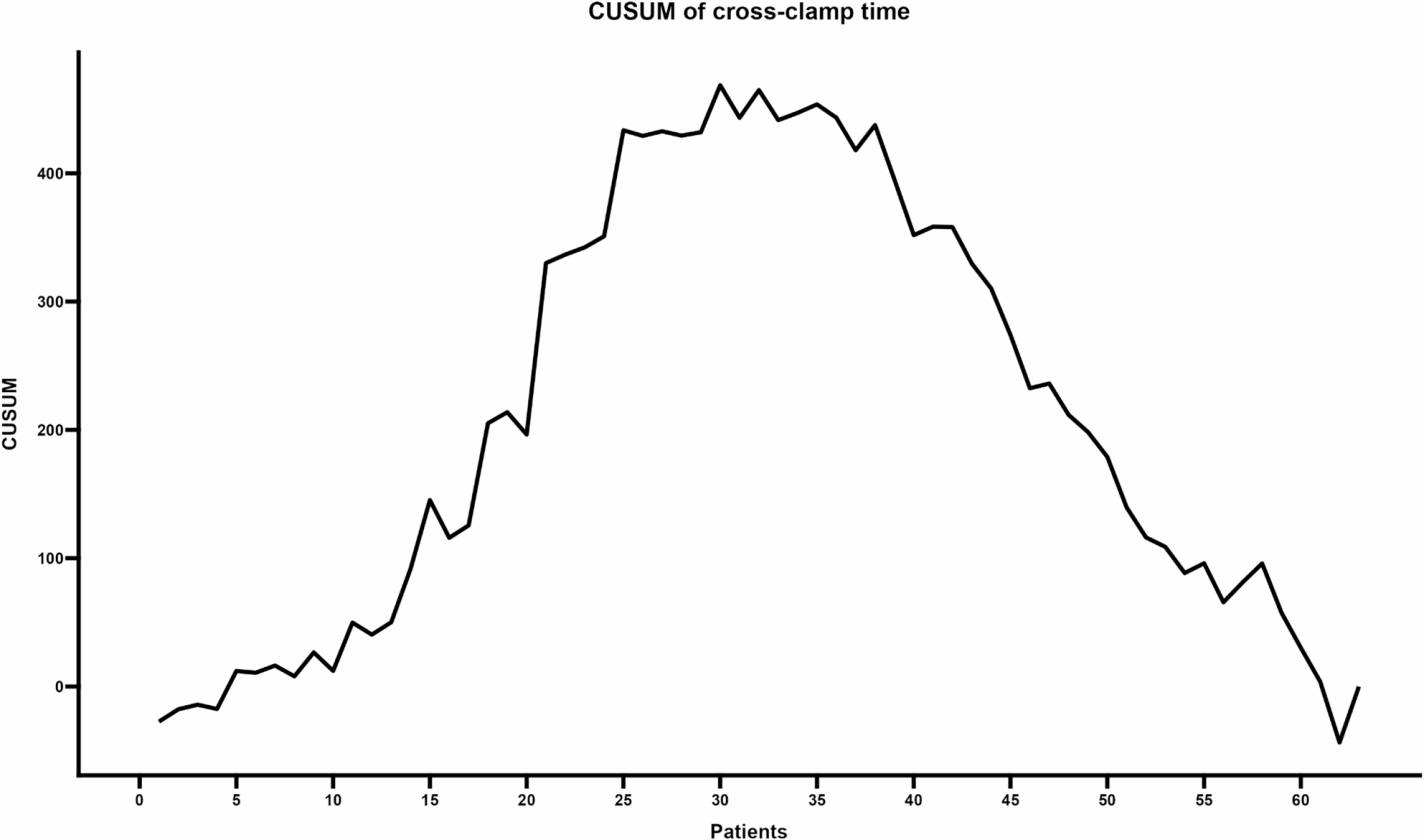
CUSUM analysis of aortic cross-clamp time. The curve indicates a stabilization phase between cases 25 and 40, followed by a progressive decline. A pronounced reduction in cumulative values is evident after case 60, reflecting increased surgical experience

**Fig. 2:**
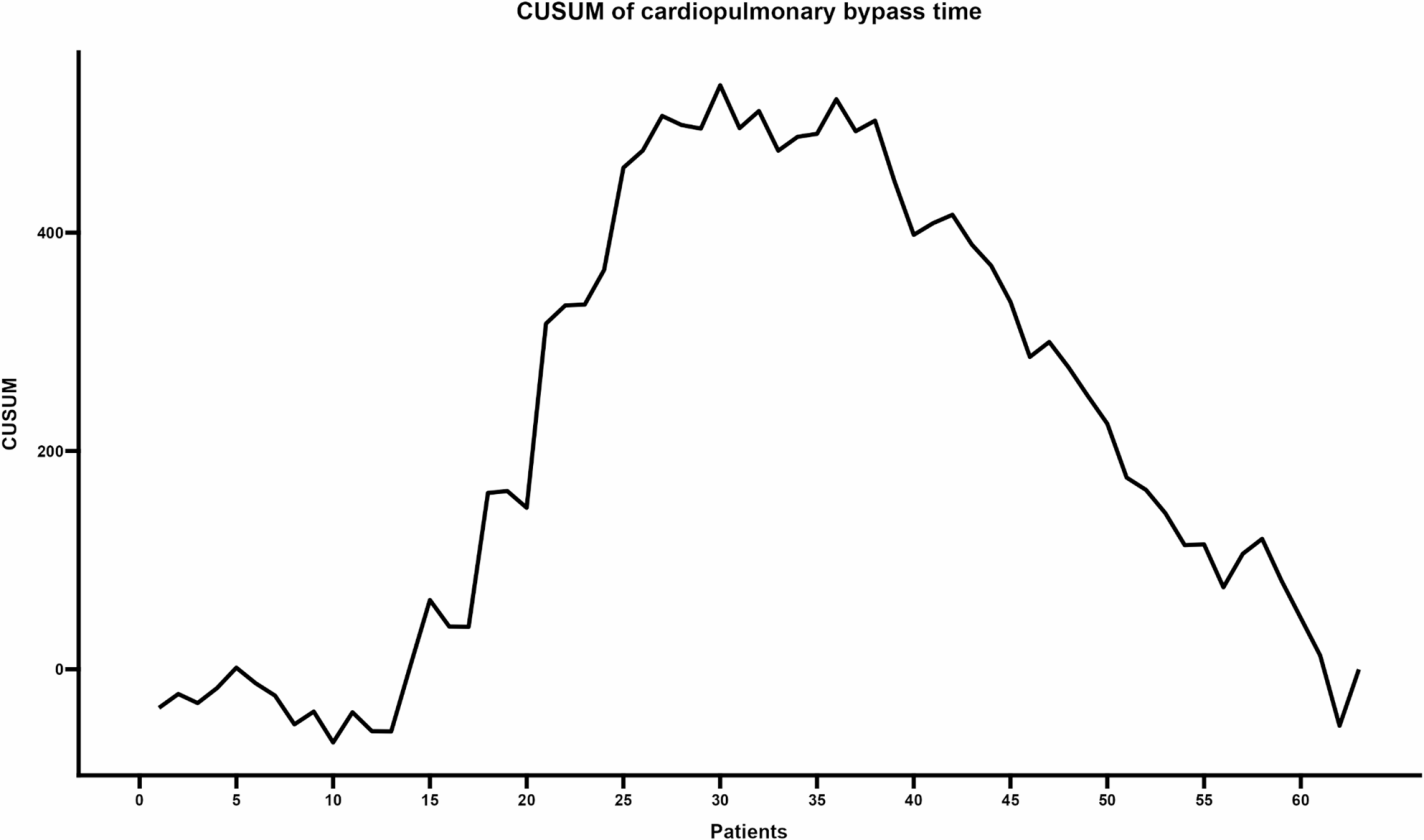
CUSUM analysis of CPB Time. The trajectory shows a peak deviation between cases 30 and 35, followed by a consistent downward trend indicating procedure times falling below the reference mean

The CUSUM analysis revealed distinct learning phases across the evaluated metrics. For cross-clamp time (Fig. 1), the curve peaked and began to stabilize between cases 25 and 40, indicating a period of maximum cumulative variability. From case 40 onward, a progressive decline was observed, with a more pronounced reduction in cumulative values evident after case 60, reflecting increased surgical experience. Similarly, CPB time (Fig. 2) showed an initial learning phase marked by an upward trajectory, peaking between cases 30 and 35. After this point, a consistent downward trend suggested that subsequent procedures increasingly recorded times below the reference mean.

**Fig. 3:**
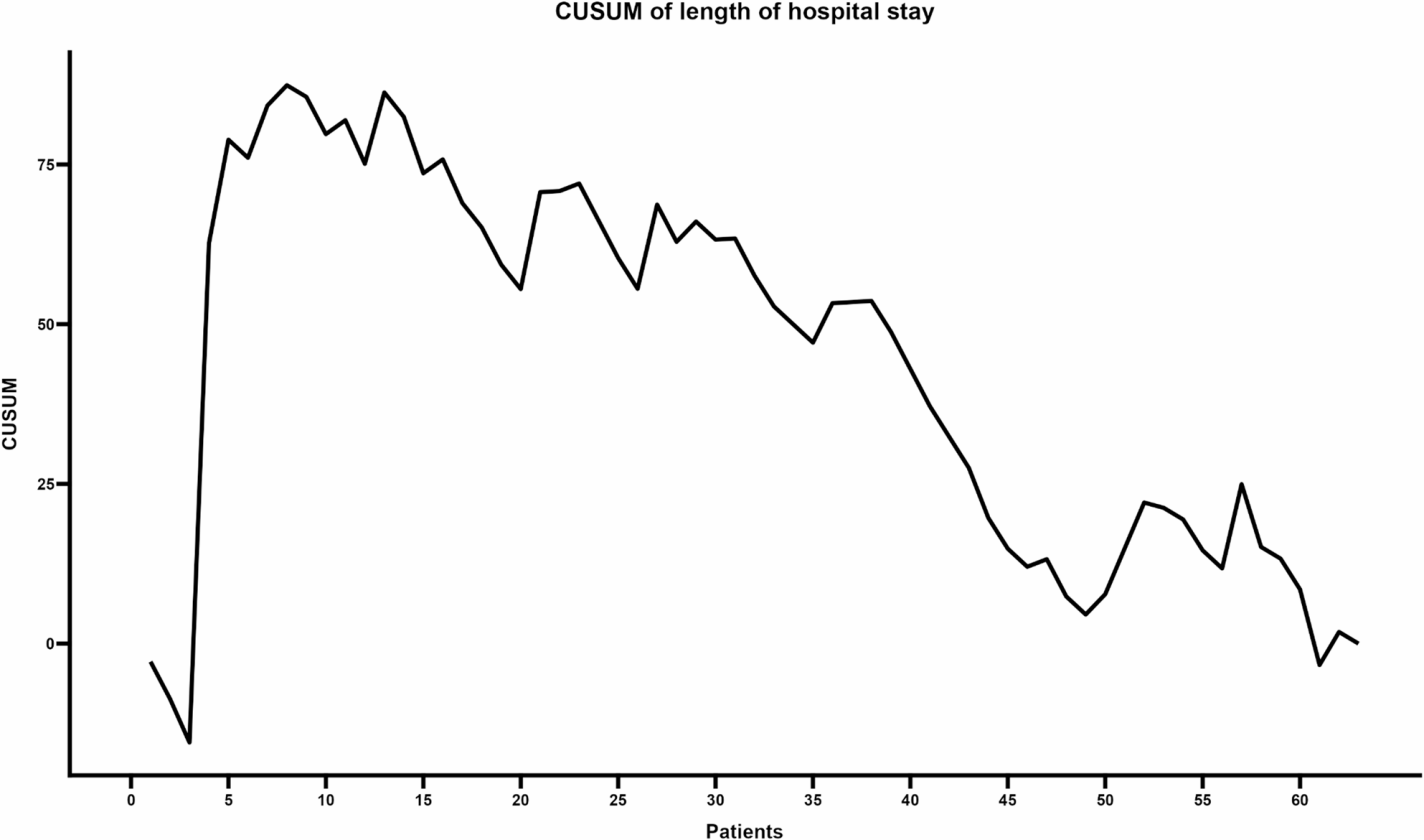
CUSUM analysis of Hospital Length of Stay. The highest accumulated deviation occurred around case 10. The curve subsequently follows a downward trajectory, achieving stabilization beyond case 49

Regarding hospital length of stay (Fig. 3), the curve initially exhibited a progressive upward slope, reaching its highest cumulative deviation around case 10. This peak marked a turning point; thereafter, a consistent downward trajectory was observed, indicating that subsequent patients increasingly experienced hospital stays below the reference mean. This trend continued to decline, with only minor fluctuations, and achieved clear stabilization beyond case 49.

**Fig. 4:**
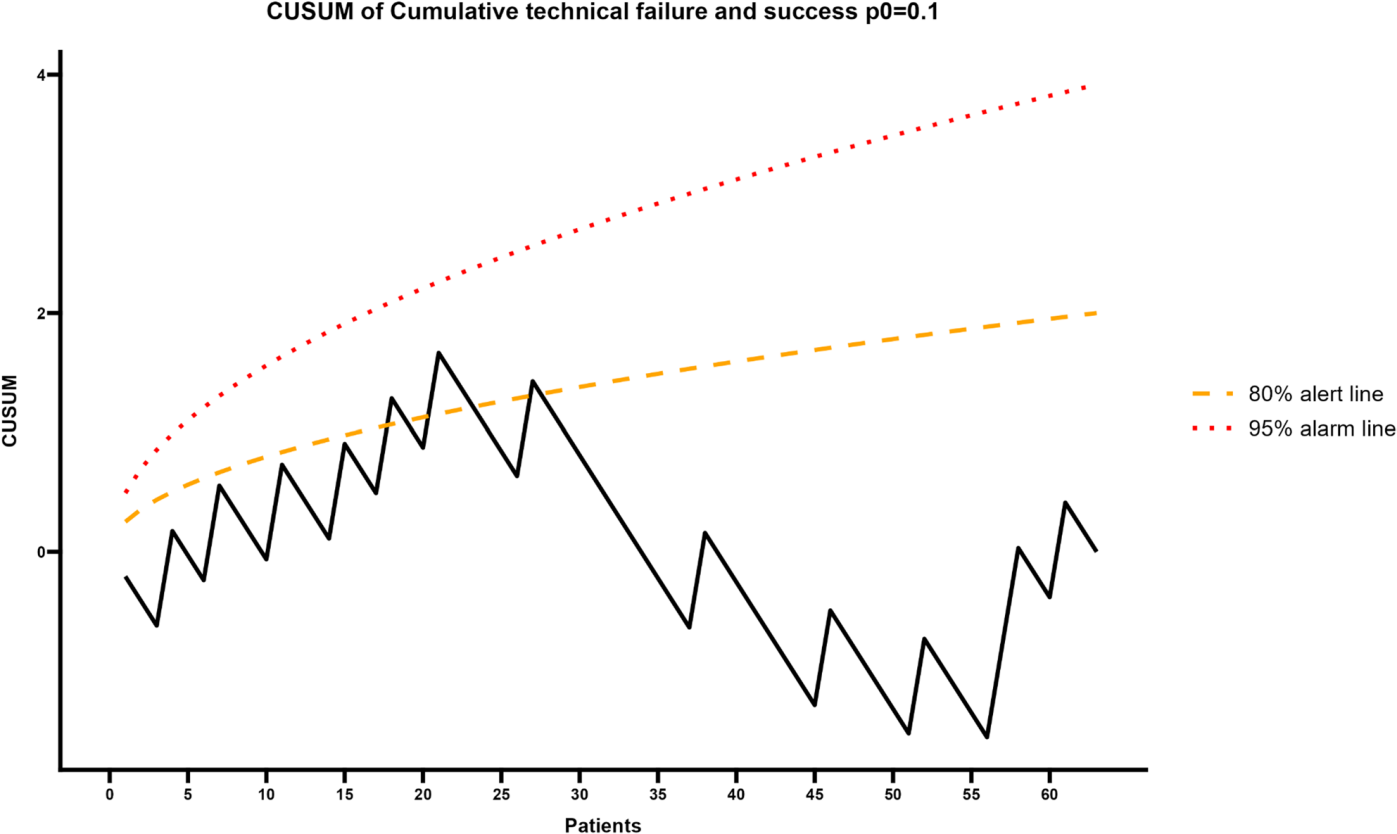
CUSUM analysis of success and failure. The plot identifies an initial learning phase peaking at case 18 (approaching the 80% alert threshold). A sustained downward trend from case 23 onward marks the consolidation of the technique

Finally, the analysis of technical success and failure (Fig. 4) revealed an early phase of instability, with oscillations peaking at case 18. At this stage, the curve approached the 80% alert threshold, although it did not exceed it. However, a decisive shift was observed from case 23 onward, when a sustained downward trend marked the consolidation of the technique. Crucially, no further breaches of alert limits occurred after this point, confirming the safety and reproducibility of the procedure.

## Discussion

Over the past decades, cardiac valve surgery has witnessed remarkable advancements in the adoption of minimally invasive approaches. While data from the Society of Thoracic Surgeons indicates a growing trend (23%) in MIM-VS [20], the majority of this evidence stems from high-volume centers in developed nations. The present study provides a distinct perspective: it explicitly demonstrates the feasibility, reproducibility, and safety of establishing a MIM-VS program in a low-volume center within a middle-income country. Despite a caseload of approximately 12 patients per year, our propensity scorematching analysis confirms that MIM-VS can be performed with outcomes comparable to those of conventional sternotomy. This reinforces the notion that high institutional volume is not an absolute prerequisite for success, provided that strict patient selection and standardized protocols are maintained. Recent literature corroborates this finding; Albåge et al. [13] showed that MIM-VS is a safe alternative in non-high-volume centers, emphasizing that favorable outcomes depend less on institutional volume and more on limiting the procedure to a small, dedicated surgical team, thereby ensuring consistent accumulation of experience.

Regarding specific clinical outcomes, no significant differences were observed between the groups in terms of intraoperative death, reoperation for any cause, transfusion requirements, and overall hospital stay. Given the lack of statistical significance in these hard endpoints, our results support a conclusion of non-inferiority regarding safety during the implementation phase. However, a statistically significant reduction in ICU stay was observed (*p* = 0.045). This finding is consistent with prior literature; Dieberg et al. [21] conducted a systematic review and meta-analysis, demonstrating a significant reduction in ICU stay of 0.7 days (*p* = 0.009) in patients undergoing minimally invasive surgery compared to MS. This finding is clinically relevant because prolonged ICU stay has been associated with an increased risk of complications and postoperative mortality, and its reduction is crucial for resource optimization in limited-resource settings.

A primary challenge of MIM-VS is its technical complexity, which leads to longer operative times. In our series, CPB time was 48 min longer and aortic cross-clamp time was 34 min longer in the MIM-VS (*p* < 0.001). These findings are consistent with a meta-analysis including matched and randomized studies, which reported an average increase in aortic cross-clamp time of 20.7 min (*p* = 0.001) and in CPB time of 36.8 min (*p* = 0.001) for minimally invasive procedures compared to conventional sternotomy [4]. The prolongation of these times may be attributed to the limited anatomical exposure in MIM-VS, which requires greater surgical dexterity. Nevertheless, in line with Kirmani et al. [12] and De Praetere et al. [22], the prolonged cross-clamp times in our study did not translate into increased mortality, morbidity, or extended hospital stay, indicating that the early phase of implementing the technique can be achieved safely despite longer procedural duration.

Focusing on the learning curve, the CUSUM analysis revealed a distinct multi-phase trajectory. A notable pattern was the oscillation in operative times: initially decreasing, then increasing, peaking between cases 25 and 40, before ultimately stabilizing in a downward trend. This trajectory aligns with the learning phases described by Holzhey et al. [23], reflecting the natural progression from initial implementation to consistent procedural performance. During this intermediate phase, variability in anatomical exposure and intraoperative complexity may temporarily prolong operative times before full technical consolidation is achieved. Our data suggest that stabilization occurs around 50 cases. While technical proficiency, reflected by improvements in CPB times, was attained earlier, the stabilization of hospital length of stay around case 49 indicates that systemic efficiency consolidates slightly later. This is consistent with findings by Papadopoulos et al. [24], who reported that a minimum of 55 procedures was required to stabilize aortic cross-clamp times. Accordingly, 50 procedures are considered to represent the threshold for program maturity in our setting. With 63 procedures performed, our results likely reflect the consolidation phase, during which further improvements in intraoperative efficiency are expected as case volume increases. These findings underscore the importance of structured training and continuous performance monitoring in optimizing the implementation of minimally invasive surgery.

The significantly higher prevalence of AF in the MS group (48.9%) must be interpreted within the context of a middle-income country. In our cohort, patients with AF were excluded from the MIM-VS group and treated via MS due to resource constraints. Unlike high-income centers where concomitant cryoablation or radiofrequency ablation is standard during MIM-VS, our institution currently lacks the consumable probes required for minimally invasive ablation. Therefore, to ensure complete treatment (Maze procedure), MS remains the standard of care for these patients in our context. This highlights a critical ‘real-world’ barrier in global cardiac surgery: the adoption of MIM-VS in emerging economies is often limited not by surgical skill, but by the availability of essential technologies [25]. Nevertheless, analysis of the distribution of AF history revealed no statistically significant association between AF and any of the evaluated outcomes (data not shown).

In a broader context, this study offers valuable insights for cardiovascular programs in Colombia and other middle-income countries, where access to minimally invasive cardiac surgery is still developing. The demonstration of a manageable learning curve, favorable clinical outcomes, and procedural safety reinforces the feasibility of adopting MIM-VS in comparable health systems, even within resource-constrained environments, particularly when institutional support, structured surgical training, and multidisciplinary collaboration are prioritized from the outset.

Our study has several limitations inherent to its retrospective, single-center design. First, the non-randomized allocation of the surgical approach was left to the surgeon’s discretion, introducing a selection bias that may have influenced the observed outcomes. Second, the single-center design may limit the generalizability of the findings to settings with differing infrastructure, surgical expertise, or patient populations. Third, the relatively small sample size may have limited the statistical power to detect significant differences in certain clinical outcomes, underscoring the need for larger studies to confirm these findings. Importantly, follow-up was limited to the perioperative period. This limitation stems from the fact that many patients reside in remote areas of the country with limited connectivity, compounded by the absence of a centralized medical record system to track outcomes beyond our institution. Finally, although safety is demonstrated, the relatively low procedural volume means that our results rely on a small, dedicated surgical team, a characteristic typical of non-high-volume centers.

## Conclusions

This study provides strong evidence that MIM-VS is a feasible and safe strategy in low-volume centers. After propensity score matching, MIM-VS demonstrated non-inferiority to MS in terms of mortality and major complications, with significant benefits in ICU length of stay. Although proficiency is typically achieved after approximately 50 cases, the procedure can be safely adopted without compromising patient safety, provided that institutional commitment and rigorous patient selection protocols are in place.

## Supplementary Information

Supplementary Material 1.

Supplementary Material 2.

## Data Availability

The datasets used and/or analysed during the current study are available from the corresponding author on reasonable request.

## Abbreviations

MIM-VS: Minimally Invasive Mitral Valve Surgery
MS: Median Sternotomy
CUSUM: Cumulative Sum
CPB: Cardiopulmonary Bypass
CABG: Coronary Artery Bypass Graft
ICU: Intensive Care Unit
TEE: Transesophageal Echocardiography
BMI: Body Mass Index
COPD: Chronic Obstructive Pulmonary Disease
AF: Atrial Fibrillation
IQR: Interquartile Range
LVEF: Left Ventricular Ejection Fraction
