# Supplementary material for: Minimally invasive mitral valve surgery in a middle-income country: feasibility and learning curve in a low-volume center
Source: J Cardiothorac Surg. 2026 Feb 28;21:155. doi: 10.1186/s13019-026-03879-3 (PMC13059528; doi:10.1186/s13019-026-03879-3)
Supplement: Supplementary file 1 — Supplementary Material 1. [file 13019_2026_3879_MOESM1_ESM.docx]

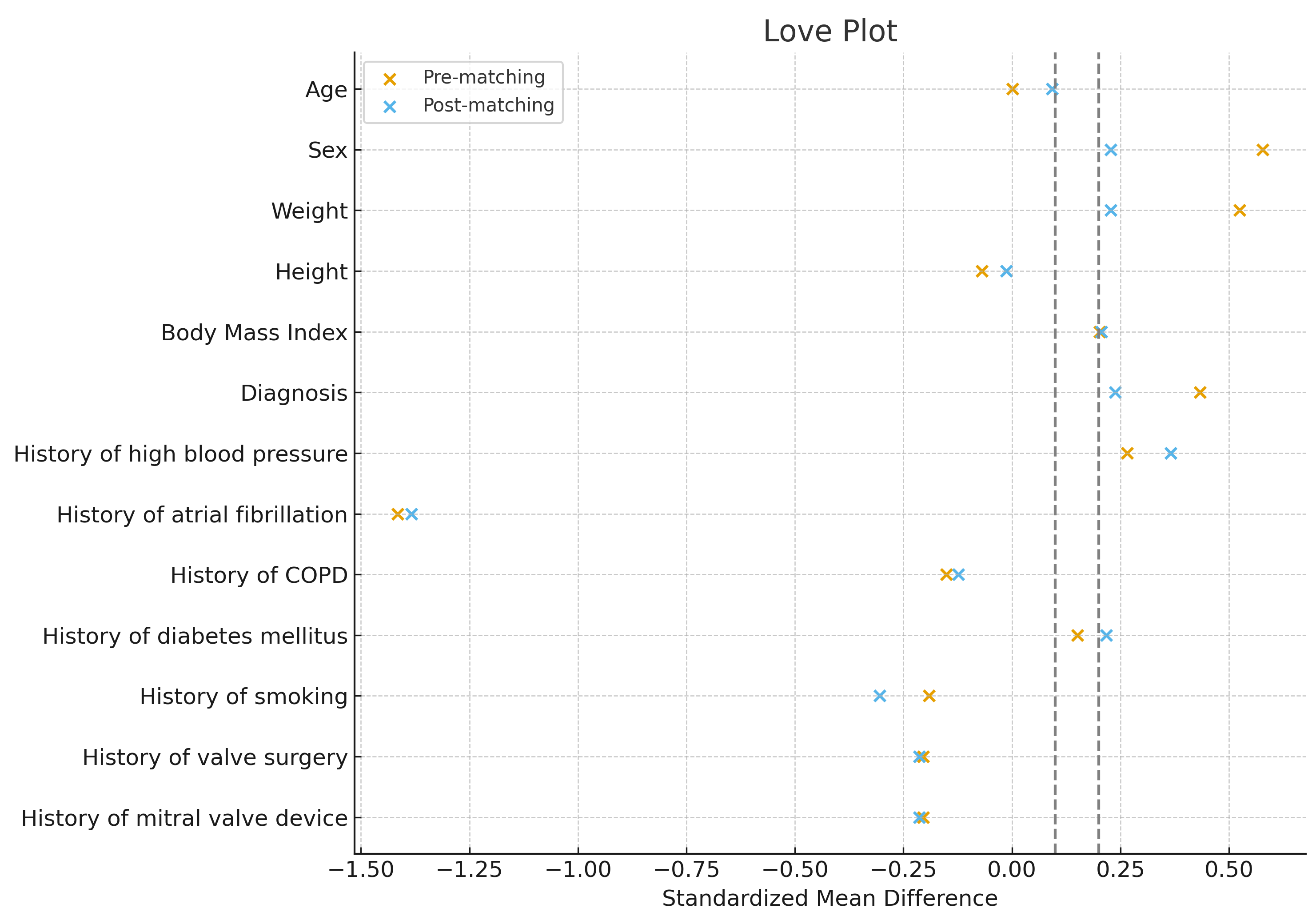


**Supplementary file 1.** Love plot: pre-matching and post-matching

Before matching, several covariates exhibited substantial imbalance between the MS and MIM-VS groups. Notably, sex, weight, BMI, diagnosis, and especially history of AF presented SMDs well above conventional thresholds, indicating clinically relevant baseline differences between groups. After matching, most variables exhibited a marked reduction in SMDs, demonstrating that the propensity score matching effectively improved comparability between groups. Continuous variables such as age, height, and weight achieved SMDs at or below the 0.1 threshold, indicating excellent balance. Likewise, categorical clinical variables, including diagnosis, history of COPD, and history of diabetes mellitus, also showed notable improvement.
