# Supplementary material for: Minimally invasive mitral valve surgery in a middle-income country: feasibility and learning curve in a low-volume center
Source: J Cardiothorac Surg. 2026 Feb 28;21:155. doi: 10.1186/s13019-026-03879-3 (PMC13059528; doi:10.1186/s13019-026-03879-3)
Supplement: Supplementary file 2 — Supplementary Material 2. [file 13019_2026_3879_MOESM2_ESM.docx]

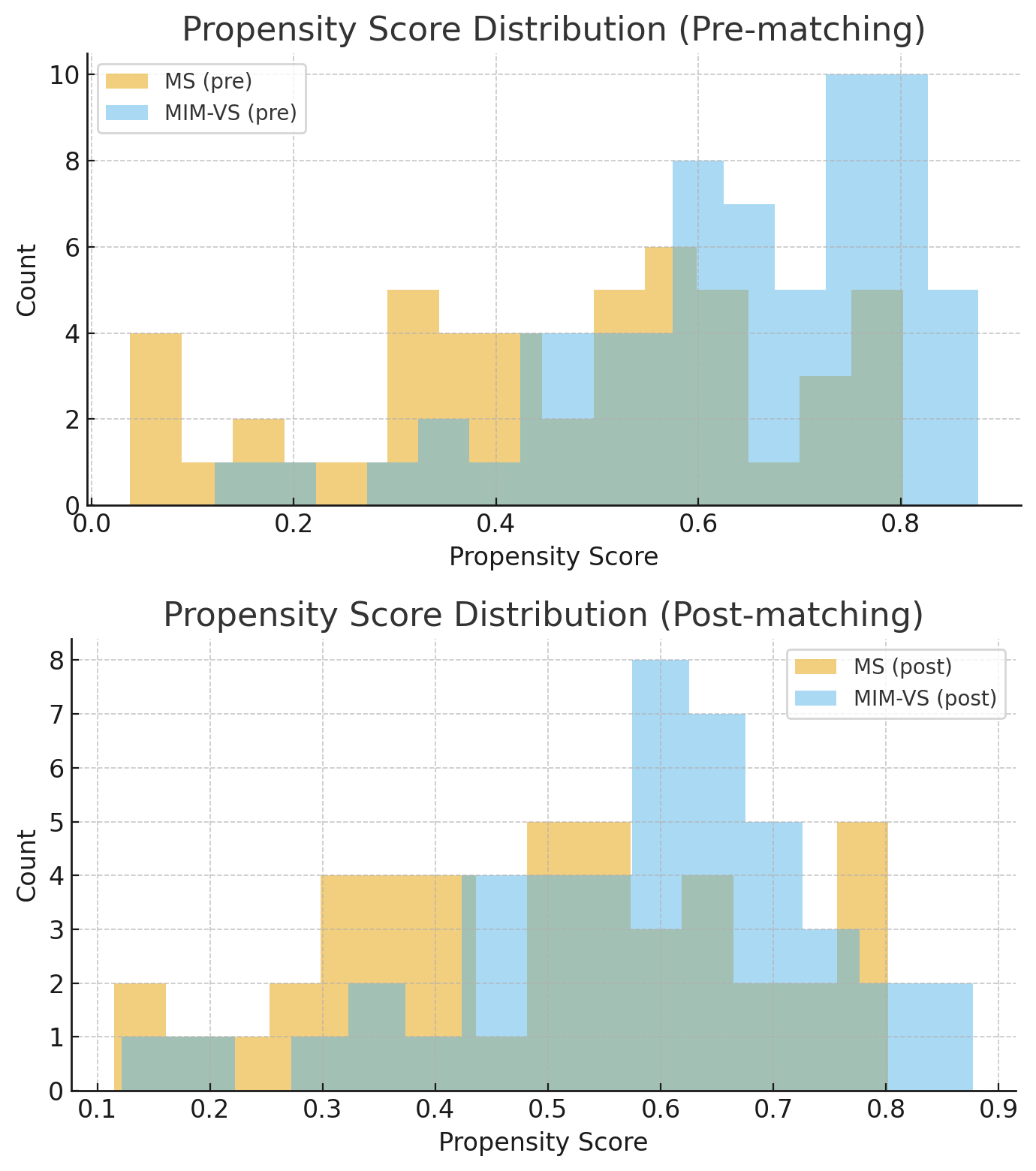


**Supplementary file 2.** Propensity score distribution: pre-matching and post-matching

Visual inspection of the propensity score distributions confirmed improved overlap between groups after matching, with most observations falling within a comparable range for both MS and MIM-VS groups. This supports the validity of the matched sample and suggests that the estimation of treatment effects is less prone to bias from extreme extrapolation.
